# Genetic Variation in the *Mcp-1* Gene Promoter Associated with the Risk of Polycystic Ovary Syndrome

**DOI:** 10.1371/journal.pone.0123045

**Published:** 2015-04-22

**Authors:** Lan Li, Ji Eun Ryoo, Kyung-Ju Lee, Bum-Chae Choi, Kwang-Hyun Baek

**Affiliations:** 1 Department of Biomedical Science, CHA University, Bundang CHA Hospital, Gyeonggi-do, Republic of Korea; 2 Hankuk Academy of Foreign Studies, Yongin, Republic of Korea; 3 Department of Gynecology and Obstetrics, CHA University, CHA General Hospital, Seoul, Republic of Korea; 4 Department of Obstetrics and Gynecology, CL Women’s Hospital, Gwangju, Republic of Korea; University of California, Los Angeles, UNITED STATES

## Abstract

Monocyte chemoattractant protein-1 (MCP-1) is a pivotal chemokine in the inflammatory response, which plays an important role in recruiting monocytes to sites of injury and infection. However, the exact mechanism of *Mcp-1* associated with PCOS risk was unknown. In this study, we explored whether the *Mcp-1* -2518G>A polymorphism increases the risk of PCOS. We performed a comparative study of -2518G>A polymorphism of the *Mcp-1* gene with PCOS. In addition, luciferase reporter assay was performed to evaluate the *Mcp-1* transcriptional activity. A strong association was observed between the -2518G>A polymorphism of *Mcp-1* gene and PCOS (*p*-value = 0.016, odd ratio (OR) = 0.693). A *p*-value under 0.05 is considered statistically significant. The genotype and allelic frequencies were assumed to be in Hardy-Weinberg equilibrium (HWE). The luciferase assays in 2 cell lines showed that the *Mcp-1* -2518G>A substitution can increase the expression of Mcp-1. MCP-1 levels in serum for PCOS group were significantly higher than those in serum for controls (*p*-value = 0.02). Furthermore, the patients carrying a genotype A/A had significantly increased levels of MCP-1 in serum compared with levels of the MCP-1 of the patients with genotypes G/G and G/A (*p*-value = 0.031). This is the first study on the genetic variation of the *Mcp-1* gene and PCOS. This finding suggests that the *Mcp-1* -2518G>A polymorphism is associated with PCOS risk by affecting transcriptional activity, leading to an increased expression level of *Mcp-1*.

## Introduction

Polycystic ovary syndrome (PCOS), a very common endocrine disorder that causes up to 30% of infertility, affects 4 to 8% of the women [1,2]. It is often accompanied with symptoms like ovarian dysfunction, androgen excess, obesity, and insulin resistance (IR) [1,3–6]. Although it is a common reproductive disease, its etiology is still not fully understood. It is now believed that PCOS is caused by combination of genetic and environmental factors[2]. Familial aggregation of PCOS indicates that genetic factors play an important role in its pathogenesis [7,8]. Up to now, the genetic research of PCOS focused on various types of genes responsible for symptoms manifest in PCOS patients. It is reported that PCOS patients tend to show characteristics of obesity, cardiovascular disease (CVD), type 2 diabetes (T2DM), and reproductive failure [9–12]. A shared genetic background among these diseases or symptoms indicates common biological pathways underlying their etiology.

Recently, several studies suggest that women with PCOS have altered circulatory levels of multiple markers of inflammation, which might also reflect a state of chronic low grade inflammation [13,14]. Additionally, chronic inflammation is associated with increased risk for CVD as well as long-term complications of PCOS [15,16]. Furthermore, PCOS has major metabolic consequences related to IR [17]. It is now evident that subclinical inflammation and IR are important predictors of CVD [18,19].

Adipose tissues are human body's major endocrine organ responsible for secretion of hormones with roles of metabolism, reproduction, and cardiovascular function [20]. It is now realized that the decrease in the level of adiponectin secreted solely by adipocytes is highly associated with obesity, diabetes, and atherosclerosis[21]. Monocyte chemoattractant protein-1 (MCP-1), a secretary factor of adipocytes, is one of the important members of C-C chemokine family, consists of 76 amino acids, and plays a key role in the recruitment of monocytes/macrophage to inflammatory foci [22]. Of note, MCP-1 is also involved in the pathogenesis of atherosclerosis, diabetes and obesity related diseases [23]. Interestingly, high glucose upregulates MCP-1 expression in endothelial cells and monocytes [24]. Another study described the overlapping pathways between blood sugar regulation and inflammation in PCOS[25]. In the case of obese patients, MCP-1, which restricts the amount of insulin-stimulated glucose uptake by adipocytes, tends to be over-expressed in adipose tissues [26]. Insulin induces the secretion of substantial amounts of MCP-1 in IR 3T3-L1 adipocytes and IR obese mice [27]. Human MCP-1 (hMCP-1) transcription is controlled by two distinct regions within the 5’-flanking sequence of the gene [28]. The first element contains two NF-κB binding sites and is essential for cytokine/chemokine-dependent regulation, while the other element contains a GC box that regulates tissue-specific expression [26,29]. In addition, the variants located at position -2518 in the distal region were proposed to regulate *Mcp-1* expression [30]. However, the variant -2518G>A of *Mcp-1* and its association with PCOS had not been evaluated yet.

In this study, we conducted experiments to determine the association between the 2518G>A polymorphism MCP-1 and women with PCOS. In addition, functional assays were performed to evaluate the effect of this SNP in PCOS.

## Materials and Methods

### Subjects

A total of 375 Korean women were recruited from the Fertility Center of the CHA General Hospital in Korea for the study. Among them, 220 women had PCOS while the other 155 were healthy control subjects. Informed consent was obtained from all patients. The diagnosis of PCOS was based on the criteria proposed by the 2003 ASRM/ESHRE Rotterdam consensus. Blood samples were collected in tubes containing EDTA as an anti-clotting factor and stored at -20°C until use. Genomic DNA was extracted from the blood of PCOS patients and control women, and 100 ng/μl genomic DNA was used for PCR. Amplified products were purified using Bioneer’s AccuPrep PCR purification kit (Bioneer, Daejeon, Korea). Plasma follicle-stimulating hormone (FSH), estradiol (E_2_), luteinizing hormone (LH), prolactin (PRL), thyroid-stimulating hormone (TSH), dehydroepiandrosterone sulfate (DHEA-S), and testosterone (T) were measured with a chemiluminescent analyzer (Beckman Coulter Inc., Fullerton, CA, USA). Serum glucose and insulin levels were measured by enzymatic and chemiluminescent methods, respectively. Using radioimmunoassay (RIA) (Siemens, Los Angeles, CA, USA), total T and free testosterone concentrations were measured in serums of patients with PCOS and controls. The intra- and inter-assay coefficients of variation (CV) values were 4.0–11% and 5.9–12% for total testosterone, and 4.0–17% and 8.0–18.3% for free testosterone, respectively.

### Ethics Statement

The study was approved by the Institutional Review Boards of Fertility Center of the CHA General Hospital. Written informed consent was obtained from all participants.

### Genetic analysis

To investigate the association between the -2518G>A polymorphism of *Mcp-1* gene and PCOS, polymerase chain reaction fragment length polymorphism (PCR-RFLP) analysis was performed for all samples.

**Fig 1:**
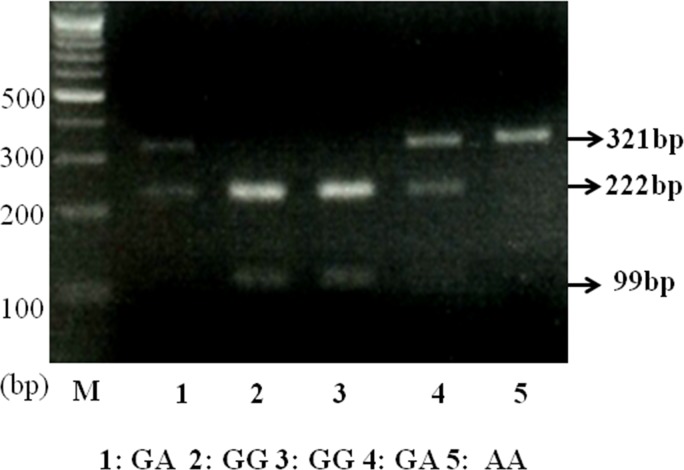
RFLP analysis of the G/A polymorphism in the promoter region of the *Mcp-1* gene.

The variant -2518G>A of *Mcp-1* was amplified using a forward primer 5’-CCG AGA TGT TCC CAG CAC AG- 3’ and a reverse primer 5’-ATC TCT GGA AAG TGA CTT GGC- 3’ by polymerase chain reaction (PCR). The cycling parameters were as follows: denaturation at 94°C for 5 min, 30 cycles at 94°C for 30 sec, 60°C for 30 sec, 72°C for 30 sec, and finally at 72°C for 5 min. Amplification products were purified using Bioneer’s AccuPrep PCR purification kit (Bioneer, Daejeon, Korea). The PCR fragment was 321 bp in length, and was digested with the enzyme *Pvu* II (New England Biolabs, Beverly, MA) for 6 hrs at 37°C. Digestion of the allele G produced two fragments with a length of 222 bp and 99 bp (Fig 1). For genotyping of SNP, the digested DNA fragments were electrophoresed on a 2% agarose gels containing ethidium bromide and visualized by an ultraviolet transilluminator.

### Cell culture

3T3L1 cells (mouse embryonic fibroblast cell line, ATCC CL-173) and OVCAR-3 (human ovarian carcinoma cell line, ATCC HTB-161) cells were cultured in Dulbecco modified Eagle medium (DMEM, Gibco-BRL) and RPMI-1640 medium supplemented with 10% FBS (fetal bovine serum, GIBCO BRL) and 1% penicillin and streptomycin (GIBCO BRL), respectively. The cells were grown at 37° C in the presence of 5% carbon dioxide in a humidified incubator.

### Construction of luciferase reporter plasmids

Reporter plasmids were constructed by inserting two lengths of the 5’-upstream region of the human *MCP-1* gene between the Kpn I and Hind III sites of the pGL3-Basic Vector (Promega Madison, WI) [28]. After cloning, all plasmids were verified by DNA sequencing.

### Transient transfection and luciferase reporter gene assay

For the reporter gene assay, 3T3L1 cells (1 x 10^5^ cells) were seeded into 2ml medium containing 10% FBS in each well of 12-well culture plate, and then cultured 24 hrs. OVCAR3 cells were plated at a density of 1.5 x 10^5^ cells per well on 12-well plates for 24 hrs. Plasmids were transiently cotransfected with 1 μg of the vector DNA containing either -2518G allele or -2518A allele by Lipofectamine 2000 (Invitrogen, Carlsbad, CA), according to the manufactures protocol. As an internal standard, all plasmids were cotransfected with 0.2 μg of pRL-SV40, which contains the *Renilla* luciferase gene to normalize the luciferase activity. 48 hrs after transfection, cells were treated with 10 ng/ml recombinant TNF-α (Peprotech Inc. Rocky Hill, NJ), and were harvested after an additional 24 hrs. Relative luciferase activity was measured with a luminometer (Infinite M200 Pro, Tecan, Salzburg, Austria) using a dual-Luciferase reporter assay system (Promega, Madison, WI) according to the instructions. For the data analysis, the firefly values were divided by the *Renilla* values, and the data are expressed as the ratio of firefly to *Renilla* luciferase activity. Independent triplicate experiments were performed for each plasmid.

### ELISA

Serum samples were collected and stored at -70°C until used. MCP-1 levels in serum were determined using a commercially available ELISA Kit (R&D Systems Inc., Minneapolis, MN, USA) according to the manufacture’s instruction.

### Statistical analysis

Statistical analysis was carried out using Hap Analysis (NGRI, Seoul, Korea; www.hap.ngri.re.kr) and GraphPad Prism 4.0 (GraphPad Software, Inc., San Diego, CA) and χ² tests were used to analyze the association between PCOS and healthy controls. Data of luciferase reporter gene assays were determined by two-way analysis of variance, and the significance of differences was determined using the unpaired *t*-test or Turkey-Kramer test. The statistical power was calculated using SPSS 12.0 software (SPSS Inc., Chicago, IL). A *p*-value < 0.05 was considered statistically significant.

## Results

### Characteristics of the study population

The analysis of the clinical and biochemical characteristics of the control subjects and PCOS cases are shown in Table 1. We followed the 2003 ASRM/ESHRE Rotterdam consensus as the diagnostic criteria of PCOS. The patients were diagnosed by polycystic ovarian morphology using ultrasonography, oligo- or amenorrhea, and clinical or biochemical hyperandrogenism (Table 1). 155 healthy control subjects did not show any PCOS syndrome. Of the 220 patients, 150 patients (68.18%) had polycystic ovaries and oligo-/amenorrhea, 25 patients (11.36%) had oligo-/amenorrhea and hyperandrogenism, 22 patients (10.00%) had polycystic ovaries and hyperandrogenism, and 23 patients (10.46%) had polycystic ovaries, oligo-/amenorrhea, and hyperandrogenism. Accordingly, we performed the analysis for specific sub-group association with quantitative traits of PCOS with the SNP -2518G>A. Clinical and metabolic features of the patients with PCOS according to the genotypes of this SNP are shown in Table 2. In addition, we did not find significant relationship between the variant in *Mcp-1* gene in obese controls and PCOS women. Body mass index (BMI), waist/hip ratio (WHR), obesity, and hormone values were considered during PCOS diagnoses (Table 1). In particular, DHEA-S level was slightly higher in the PCOS patients, whereas the concentrations of LH, T, and free T were dramatically higher in the PCOS patient group (Table 1).

**Table 1:** Clinical and biochemical profiles of 155 normal controls and 220 patients with polycystic ovary syndrome (PCOS).

Characteristics	NNormal control (n = 155)	PPCOS patient (n = 220)	***P*** value
Age (y)	31(27–42)	32(26–40)	NS
Body Mass Index (kg/m^2^)	21.82±2.62 (16.75–31.74)	23.01±4.34 (17.02–36.87)	NS
Waist/hip ratio (WHR)	0.79±0.06 (0.70–0.90)	0.80±0.07 (0.67–1.13)	NS
Obesity^a^	n = 6 (3.9%)	n = 12 (5.45%)	
Polycystic ovaries and oligo- or amenorrhea	n = 0 (0.00%)	n = 150 (68.18%)	
Polycystic ovaries and hyperandrogenism	n = 0 (0.00%)	n = 22 (10.00%)	
Oligo- or amenorrhea and hyperandrogenism	n = 0 (0.00%)	n = 25 (11.36%)	
Polycystic ovaries, oligo- or amenorrhea and hyperandrogenism	n = 0 (0.00%)	n = 23 (10.46%)	
FSH levels (mIU/ml)	6.52±2.62 (3.33–17.72)	6.21±5.87 (3.24–25.19)	NS
LH levels (mIU/ml)	3.42±2.29 (1.05–7.10)	7.63±5.63 (1.16–29.96)	<. 001
Prolactin (ng/ml)	12.96±6.17 (4.04–35.34)	12.92±7.61 (4.19–34.64)	NS
E2 (pg/ml)	33.25±19.49 (4.33–62.25)	39.02±23.06 (2.28–119.61)	NS
TSH (μIU/ml)	2.29±1.19 (0.30–5.37)	2.29±1.13 (0.65–5.49)	NS
DHEA-S (μg/dl)	154.79±59.44 (53.63–296.93)	210.14±81.62 (53.43–401.10)	0.01
Total testosterone (ng/ml)	0.26±0.13(0.06–0.66)	0.58±0.40 (0.09–3.61)	<. 001
Free testosterone (pg/ml)	0.53±0.12 (0.38–0.60)	0.88±0.60 (0.73–0.90)	<. 001
Insulin (μIU/ml)	12.44±6.38 (4.02–23.25)	13.05±3.75 (5.36–24.41)	NS
Fasting glucose	90.54±10.05 (75.00–126.00)	93.42±13.07 (71.10–136.00)	NS

Numerical data were presented as means ± standard deviation (SD) or n (%);

NS not significant.

Abbreviations: BMI, Body mass index: WHR, Waist-hip ratio: FSH, Follicle-stimulating hormone: LH, Luteinizing hormone: E2, Estradiol: TSH, Thyroid-stimulating hormone; DHEA-S, Dehydroepiandrosterone-sulfate.

^a^ Body mass index (BMI) ≥25 kg/m^2^.

**Table 2:** Clinical features of women with polycystic ovary syndrome (PCOS) according to the genotypes.

Polycystic ovaries & oligo- or amenorrhea			Polycystic ovaries & hyperandrogenism			Oligo- or amenorrhea & hyperandrogenism			Polycystic ovaries, oligo- or amenorrhea &hyperandrogenism		
n = 150			n = 22			n = 25			n = 23		
G/G	G/A	A/A	G/G	G/A	A/A	G/G	G/A	A/A	G/G	G/A	A/A
66 (44%)	60 (40%)	24 (16%)	11 (50%)	8 (36.4%)	3 (13.6%)	14 (56%)	7 (28%)	4 (16%)	14 (60.9%)	6 (26.1%)	3 (13%)

### Association between the MCP-1 -2518G>A polymorphism and PCOS risk

For our study, the frequencies of G/G, G/A, and A/A genotypes of -2518G/A polymorphism in *MCP-1* gene was analyzed by RFLP analysis and the result showed different proportion (*P*-value = 0.016, odd ratio (OR) = 0.693) between PCOS and control groups. The frequencies of GG, GA, and AA genotypes were 102 (46.3%), 84 (38.2%), and 34 (15.5%), among the cases, and 57 (36.8%), 62 (40%), 36 (23.2%), among the controls, respectively. Regarding the X^2^ test, both groups were in agreement with the Hardy-Weinberg equilibrium (HWE, *p*-value = 0.21) (Table 3).

**Table 3:** Allele frequencies of G/A polymorphisms of the *MCP-1* gene in controls (n = 155) and polycystic ovary syndrome (PCOS) patients (n = 220).

Characteristics	Control (n = 155)	PCOS (n = 220)
2518G/A		
GG	57 (36.8%)	102 (46.3%)
GA	62 (40%)	84 (38.2%)
AA	36 (23.2%)	34 (15.5%)
Alleles		
G	176 (56.8%)	288 (65.5%)
A	134 (43.2%)	152 (34.5%)
**Allele frequency**	**OR(95% CI) = 0.693(0.514–0.934)**	***p*-value = 0.016**

*CI*, confidence interval; *OR*, odds ratio

### Effect of the MCP-1 -2518G/A polymorphism on transcriptional activity

**Fig 2:**
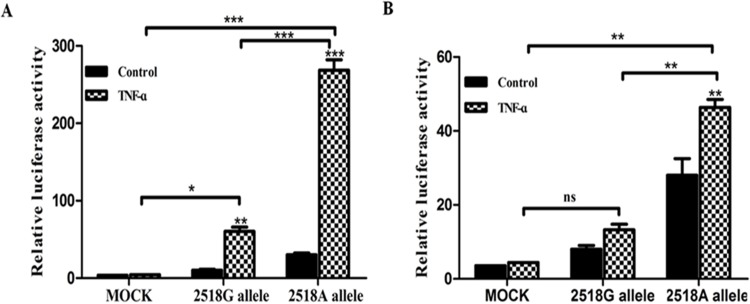
Two constructs were transiently transfected into 3T3L1 (A), and OVCAR3 (B) cell lines, respectively. (A) TNF-α induced a significant increase in luciferase activity from all the constructs. Moreover, the transcription activity of AA constructs was approximately 5 fold greater than the activity of GG constructs in 3T3L1 cells. (B) The OVCAR3 cells transfected with -2518A allele had a 3 fold increase in reporter gene activity as compared with the -2518G allele. Luciferase activity of each construct was normalized against internal control of *Renilla* luciferase. Each experiment was performed three times. Data indicate a mean value with SEM from 3 independent experiments. *P < 0.05, **P < 0.01, ***P < 0.001. ns, not significant. Data were analyzed by a two-way analysis of variance, and significant differences were determined by Tukey-Kramer test.

To investigate whether the *Mcp-1* -2518G>A polymorphism is associated with the *Mcp-1* transcriptional activity; we generated luciferase reporter vectors by using pGL3-basic vector with either -2518G or -2518A allele. Then, we used them for transfection with the 3T3L1 and OVCAR3 cells. As shown in Fig 2, the -2518A allele had a significant increase in the relative luciferase activities, compared to the one that with the -2518G allele in these two cell lines (two-way ANOVA, F_1, 6_ = 386.81, p< 0.0001 Alleles; F_2, 6_ = 324.40, p< 0.0001 TNF-α treatment; F_2, 6_ = 218.02, p< 0.0001 Alleles*TNF-α treatment, and F_2, 6_ = 132.42, p< 0.0001 Alleles; F_1, 6_ = 21.39, p = 0.0036 TNF-α treatment; F_2, 6_ = 8.97, p = 0.0158 Alleles*TNF-α treatment, respectively). These results suggest that the -2518A allele in the promoter region is associated with an increased transcriptional activity of the *Mcp-1* gene.

### ELISA

**Fig 3:**
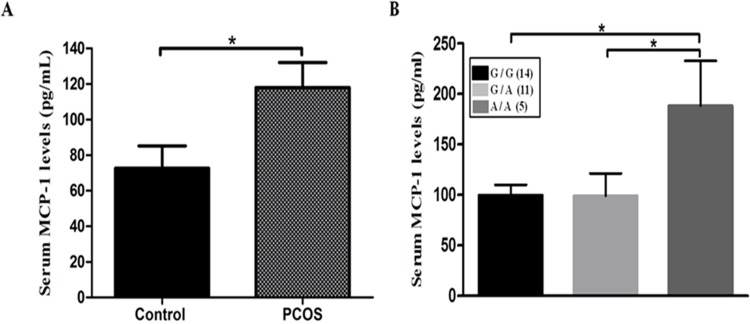
MCP-1 levels in the serum of controls (n = 25) and PCOS patients (n = 30) were quantified by ELISA (mean ± SEM of duplicate wells). A, 72.64 ± 12.58 pg/ml in controls; 118.0 ± 14.11 pg/ml in PCOS patients. *P< 0.05. Data were analyzed by unpaired *t*-test. B, Serum MCP-1 levels in women with PCOS according to the genotypes of SNP -2518G>A. Data are presented as means ±SEM.

We further measured serum MCP-1 levels of the freshly collected blood samples of control subjects (n = 25) and patients with PCOS (n = 30). Interestingly, the MCP-1 levels were significantly different between PCOS patients and controls (72.64 ± 12.58 pg/ml vs 118.0 ± 14.11 pg/ml, *P* = 0.02; Fig 3A). In addition, the patients with genotypes G/G and G/A had significantly decreased MCP-1 levels in serum compared with those of the carriers with the genotype A/A (*P* = 0.031; Fig 3B).

## Discussion

Inflammation is considered as a normal, yet sometimes a chronic issue that causes some problems [31]. For PCOS, chronic inflammation creates IR and influences the way ovaries produce and release eggs [32]. Besides, chronic low-grade of inflammation found in obese patients is characterized by macrophage accumulation in adipose tissues and abnormal cytokine production is a key marker of obesity and T2DM [33–36].

In this study, we have investigated one of the pivotal adipocyte secretary factors, *Mcp-1*, and analyzed data on the relationship between the -2518G>A polymorphism of *Mcp-1* and Korean women with PCOS. Our study demonstrated that the -2518G>A polymorphism of *Mcp-1* is associated with PCOS. After identifying the correlation between the gene polymorphism and PCOS, we further analyzed to better understand the susceptibility of the *Mcp-1* gene variation in the development of PCOS. Further luciferase reporter assay showed that the *Mcp-1* -2518G>A substitution can increase the transcription activity of the *Mcp-1* gene in vitro. Additionally, after cells were treated with TNF-α (10 ng/mL), the -2518G>A allele showed a significant increase in the relative luciferase activities, compared to those with the -2518G allele in 3T3L1 and OVCAR3 cell lines (all *P*<0.05). These data suggest that the -2518G in the promoter region is associated with an increased transcriptional activity of the *Mcp-1* gene. To support the in vitro data, we performed ELISA for the quantitative assay of MCP-1, to demonstrate that the patients with the *Mcp-1* polymorphism have increased MCP-1 levels. This profile suggests that they may present a lower degree of chronic inflammation than usually seen in PCOS from other ethnicities [37–39].

There was no substantial research showing the correlation between the variant of *Mcp-1* and PCOS. Only a small case-control study in China has reported that PCOS patients tend to show high *Mcp-1* levels, increasing the risk of atherogenesis [40]. One study conducted in Turkey showed that lean and young PCOS patients have high *Mcp-1* levels, which can serve as the early indications of cardiovascular disease and vascular damage [37]. Furthermore, several studies have also demonstrated that women with PCOS have elevated MCP-1, which was verified in PCOS and control subjects after adjustment for age and body mass index [13,38,40,41]. Another study performed at Denmark University Hospital indicated that plasma *Mcp-1* and macrophage inflammatory protein-1 α are increased in patients with PCOS and associated with adiposity [38]. Furthermore, the variant of *Mcp-1* can be a future predictor for long-term kidney graft failure [42]. *Mcp-1* AA genotype and A allele have been proposed to play a specific role in determining diabetic susceptibility (DN), but they do not seem to be important in clinical manifestations of DN in a Turkish population [43].

In conclusion, the A allele of *Mcp-1* SNP rs1024611 is associated with higher levels of MCP-1 and a protective factor for PCOS. Also, we have identified a functionally significant -2518G>A polymorphism in the *Mcp-1* promoter region, which may contribute to the development of PCOS. However, the findings in the present study were only from a Korean population. Therefore, further validation of the variant of *MCP-1* in *vivo* and larger studies with various ethnic backgrounds are required for a better understanding of pathogenesis for PCOS.
